# The effectiveness of health care finance in promoting health: does the condition of health get better by spending more?

**DOI:** 10.11604/pamj.2022.42.95.35133

**Published:** 2022-06-06

**Authors:** Nasreddine Aissaoui, Lamia Hamaizia, Said Khalfa Mokhtar Brika, Ahmed Laamari

**Affiliations:** 1 Faculty of Economics, Business and Management Sciences, Oum El Bouaghi University, Oum El Bouaghi, Algeria,; 2 Department of Administrative Sciences, Applied College, University of Bisha, Bisha, Saudi Arabia,; 3 Faculty of Science and Art in Al-Namas, University of Bisha, Bisha, Saudi Arabia

**Keywords:** Health spending, health care finance, efficiency, US health system

## Abstract

Medical research in the United States remains a global reference, endowed with unrivalled financing, a source of endless advancements, and recognized with many accolades; with 45 per cent of the winners, the United States outrageously dominates the Nobel Prize for Medicine. The volume of health spending in the United States is far more than any other country; however, the health outcomes are far below expectation. An American child Born in 2016 will live on average 78.6 years, which places the country around the thirty-fifth place in the world, somewhere between Cuba and Qatar; the United States has other modest results, as evidenced by the ranking of countries in terms of infant mortality in 2015, which placed the country 33^rd^ out of 35 member countries, ahead of only Turkey and Mexico. Although the United States ranks 35th out of 190 countries based on infant mortality in 2015, it is still far behind Cuba, which was 30^th^ and the first “non-high” income country. In 2016, US health expenditures/gross domestic product (GDP) exceeded 16%, with an average of 10,000 USD/inhabitants, while Cuban health expenditures/GDP did not exceed 11% during the same period. We aim through the present work to show that the state of health doesn't improve by spending more. However, it improves by spending more on programs that we know from the evidence can improve health outcomes.

## Opinion

We will first provide proof of our hypothesis by comparing the American healthcare system to that of Cuba, then to that of Spain. Several studies have focused on the characteristics and specificities of the Cuban health system, as well as its effectiveness and efficiency [1]. The Cuban government has achieved universal access to health care for all categories of the population. Despite relatively limited resources and the devastating effect of the United States economic sanctions enforced for more than a half-century.

Cuba has managed to generalize access to health care for all categories of the population, and to achieve results similar to those of the most developed countries: Cuba is the only country with a health system closely linked to research and development [2]; this small nation has built its health system around preventive medicine, the results have been extraordinary so far [3]; it has the lowest infant mortality rate (4.2% live births), it is lower than that of the United States, and it is among the lowest in the world [4]. Although Cuba's political and economic system does not converge with other countries on the continent, it remains that the effectiveness of health financing and the results achieved in Cuban public health... may be a model for Low- and Middle-income Countries.

Cuba is one of the best countries in the Americas and the Third World, with results similar to those of the most developed countries, this country recorded a life expectancy of around 78 years. This life expectancy is the highest for people over 60 years in Latin America [5]. We must not forget that Cuba has been under an embargo for 60 years, and that has not stopped that country from training doctors and then sending thousands of them to middle-income or low-income countries like Algeria, or helping high-income countries like Italy during the COVID-19 pandemic.

Spain is positioned as the third-best health system, behind the untouchables Hong Kong and Singapore, in the Bloomberg ranking of 2019 [6]. Spain reaches the top of the ranking of the healthiest countries in the world, moving from sixth place in 2017 to the first place in 2019. The Bloomberg index is based on many criteria, such as: life expectancy, risks related to alcohol and tobacco, access to drinking water, the practice of physical activity, etc. Unfortunately, the United States is not among the 20 richest countries in the world in this ranking [7].

Spain is among the countries of the European Union which will have very high life expectancy by 2040; the population of this country will even have the longest lifespan in the world, nearly 86 years [8]. Public health care providers offer primary health care to children, women and the elderly, as they offer, in the majority of cases, acute care and long-term care. The number of people dying from cardiovascular diseases and cancers in Spain has dropped significantly over the past decade.

We are now going to analyse the thesis and the antithesis, as we are going to look for the causes that place the American health care system in an uncomfortable rank, which does not always match the volume of financing of the health care system of this country.

We believe that there are some who do not agree with the idea of comparison, and there are even some who may say that it will not be fair to compare the US health system with that of Cuba because the United States has one of the most competitive and complex healthcare systems. If all things remain equal, more spending will improve health services and therefore better health. Of course, there is little evidence on the causal relationship between increased public spending and the positive impact on the health status of a population. However, when comparing the contexts of poor and rich countries, the effect of public health expenditure will be very interesting, by increasing its volume, we can clearly see the positive results on the populations of low- and middle-income countries. What will be certain is the inverse relationship between public expenditure and that of the private sector in this sector. However, what is guaranteed as results is not the case for high-income countries, in this case the USA [9, 10].

Another explanation is the ability of high-income countries to better harness additional public health spending, in order to improve their health outcomes, in ways that other countries do not. If we return to the case of the American health care system, there is another point to consider: that of government regulation. If we take the case of prescription drugs, one of the biggest drivers of overspending in this country, the United States allows market competition to dictate prices; Pharmaceutical companies and insurers are both responsible for prices, and the volume of drug expenditure. However, in countries where health expenditure is lower, there is government intervention that controls prices either directly or indirectly.

If we take the United States as an example, you can see why there is no absolute correlation 1 to 1 between health expenses and health results. The United States has an amazing excess of the quality of the health care services provided for those who are insured, which counts in this analysis. However, we believe that if we adopt a more global point of view, we will find a certain correlation between spending and results, which has different degrees. It is also possible that any correlation that you observe is not causal, because public expenditure to improve daily life: sanitation, access to food and clean water, etc. is also important to improve the state of health of the population. However, the relationship is bidirectional, and there is evidence that health care expenses have net economic benefits.

More health investments cause better quality of health care. However, the difficulty is that the organization of the health system is not the same for all countries. However, we know how prices are established in the USA; there are monopolistic pricing conditions in force [11]. What is the cost of hepatitis C antiviral therapy? Well, according to the US monopoly system, the average price is 80,000 USD; In Spain, it is around 60,000 USD for the health care provider; And in India, which does not follow this approach to determine the price, it is 130 USD [12].

It is clear that the United States has always spent more than 17% of its GDP on health, to finance a system that could simply be considered onerous and unfair. Everyone knows the reputation of notable institutions: Mayo Clinic, Mount Sinai Hospital, Cleveland Clinic, Johns Hopkins etc. But these prestigious establishments, at the highest level of quality and progress in terms of care, struggle to accommodate patients without health coverage, or with limited coverage, or who find it difficult to take charge of complex care or costly procedures, by sticking to a restrictive list of interventions and medications. Similarly, outpatients insured by the Health Maintenance Organization (HMO) are often refused reimbursement for an act or treatment deemed inappropriate or even non-essential by the insurer [11, 13].

If the answer to the question is now quite clear, what are the solutions that can make a health system more efficient? The answer to this question requires the collaboration of researchers to find solutions to the health problem, which do not involve increased expenditure. Compared to other industrialized countries, health inequalities in the United States are among the largest in the world. Therefore, we believe that investing more money would not solve the problem of health equity with a health system like the one that currently exists. We believe that as researchers; we are challenged to take this issue seriously, until we can change the way operations are conducted on it, especially around health.

We believe that health status improves by spending more on programs that we know, based on the evidence, can make a difference in health outcomes. Therefore, it is not a question of the amount of budget but a question of how these funds will be used; what are the priorities? What are the percentages allocated to each area? And how is this allocation linked to evidence, studies and outcomes for better health?

We are not saying that the American healthcare system is a bad health care system; even the best are not perfect. In Spain for instance, a situation in which the doctors are mostly public officials, hired by the government and low compensated, but who are protected from dismissal unless they commit a significant wrong. Thus, the objective, according to the Spanish national health system “Instituto Nacional de la Salud”, is to avoid making a serious error that interferes with the rules, the latter are recommendations of good practice by the WHO [7, 14]. So, the goal is not to cure everyone; rather, it is about obeying instructions and you will be a faithful servant, perhaps even a competent physician. Although the system seems less than optimal, it has proven to be so effective that Spanish enjoy one of the longest lives in the world, despite having health insurance that costs around USD 100 per person available to everyone. Because the system is not both sustainable and fair, that is the problem. When a system is in crisis, it will be considered as an obsolete system! This happened during the COVID-19 pandemic. Spain, with a population of 47 million inhabitants, one of the European countries most affected by the pandemic, recorded on Wednesday, February 24, 2021: 3161,432 cases recorded and 68,079 deaths due to the coronavirus [15]. As in other countries, this assessment is however clearly underestimated, where many victims could not be tested during the first wave of the epidemic, in the spring of 2020, due to the saturation of the health system.

The expected results can measure the performance of a health system. Otherwise: if citizens do not fully benefit from care services through demographic, financial and organizational accessibility; and if the state of health does not reflect the wonderful image of the world´s leading power so the reputation of a country cannot hide the shortcomings of a health system!
